# Human kallikrein-2 gene and protein expression predicts prostate cancer at repeat biopsy

**DOI:** 10.1186/2193-1801-3-295

**Published:** 2014-06-11

**Authors:** Raj Satkunasivam, William Zhang, John Trachtenberg, Ants Toi, Changhong Yu, Eleftherios Diamandis, Michael W Kattan, Steven A Narod, Robert K Nam

**Affiliations:** Division of Urology, Sunnybrook Health Sciences Centre, Sunnybrook Research Institute, University of Toronto, 2075 Bayview Avenue, Suite MG-406, Toronto, ON M4N 3 M5 Canada; Division of Urology, Princess Margaret Hospital, University of Toronto, Toronto, Ontario Canada; Department of Medical Imaging, Princess Margaret Hospital, University of Toronto, Toronto, Ontario Canada; Quantitative Health Sciences, The Cleveland Clinic, Cleveland, Ohio U.S.A; Department of Biochemistry, Mount Sinai Hospital, University of Toronto, Toronto, Ontario Canada; Department of Public Health Sciences, Women’s College Hospital, Women’s College Research Institute, University of Toronto, Toronto, Ontario Canada

**Keywords:** Human Kallikrein-2, Nomogram, Prostate cancer, Single nucleotide polymorphisms

## Abstract

**Purpose:**

The human kallikrein-2 (hK2) protein and two single nucleotide polymorphism (SNPs) (rs2664155, rs198977) of the gene are associated with prostate cancer risk. We examined whether hK2 protein and gene SNPs predict prostate cancer at the time of repeat biopsy.

**Methods:**

We prospectively offered a repeat biopsy to men with a negative prostate biopsy performed for a PSA >4.0 ng/mL or abnormal Digital Rectal Exam (DRE) between 2001–2005. We genotyped and measured serum hK2 levels in 941 men who underwent a repeat prostate biopsy. Logistic regression analyses were conducted to determine the significance of KLK2 SNPs and hK2 levels for predicting cancer at repeat biopsy.

**Results:**

Of the 941 patients, 180 (19.1%) were found to have cancer. The rs198977 SNP was positively associated with cancer at repeat biopsy (OR variant T allele = 1.8, 95% CI: 1.04-3.13, p = 0.049). When combined, the odds ratio for prostate cancer for patients with high hK2 levels and the variant T-allele of rs198977 was 3.77 (95% CI: 1.94-7.32, p < 0.0001), compared to patients with low hK2 levels and the C-allele. The addition of hK2 levels and KLK2 rs198977 to the baseline predictive model did not significantly increase the area under the curve from a baseline model of 0.67 to 0.69 (p = 0.6).

**Conclusions:**

The KLK2 SNP rs198977 was positively associated with hK2 levels and predicts prostate cancer at the time of repeat prostate biopsy. Further characterization of the KLK2 gene will be needed to determine its clinical utility.

## Introduction

Transrectal ultrasound guided biopsy of the prostate is the gold standard to establish the diagnosis of prostate cancer among prescreened men. Multiple needle cores are obtained randomly within targeted areas of the prostate gland, since tumour foci are too small to be visualized. Despite the multiple samplings, areas of cancer may be missed by the biopsy procedure and the false negative rate has ranged from 10% to 30% (Rietbergen et al. 1998; Durkan & Greene 1999). The practice of conducting a repeat biopsy after an initial negative biopsy is variable and not well established due to the lack of definitive predictor variables that can identify patients at high risk for having prostate cancer (Djavan et al. 2000; Roehl et al. 2002).

We and others have shown that a serine protease, human kallikrein-2 (hK2), was positively associated with prostate cancer (Klein et al. 2010; Nam et al. 2000; Herrala et al. 1997). We also showed that single nucleotide polymorphisms (SNPs) of the KLK2 gene, which encodes for the hK2 protein have been shown to be associated with prostate cancer (Nam et al. 2003; Nam et al. 2006; Nam et al. 2009).

To examine whether these factors could be used to determine the presence of cancer among patients at the time of repeat biopsy after an initial negative biopsy, we conducted a prospective study among a cohort of men who had previously undergone biopsy with no evidence of prostate cancer. We offered a repeat biopsy among these men and developed a nomogram to guide repeat biopsy in men having an initial negative biopsy. We also conducted decision curve analysis to determine whether these factors related to hK2 provided additional predictive value compared to conventional risk factors.

## Materials and methods

### Study subjects

The study subjects were drawn from a pre-screened group of men at Sunnybrook Health Sciences Centre and University Health Network (Toronto, Canada) who underwent an initial prostate biopsy for either PSA >4.0 ng/mL or abnormal DRE. After institutional research ethics board (REB) approval, we offered all men over the age of 40 whom had an initial negative biopsy a repeat biopsy over the period of 2001 to 2005. A total of 1200 consecutive men with a negative biopsy over the study period were offered a repeat biopsy within a span of 6 months to 12 months after the date of their initial biopsy. Of 1200 men, 941 (78%) agreed to undergo a repeat biopsy. All repeat biopsies followed an extended 12-core TRUS-guided needle-biopsy technique (Babaian 2000).

Baseline demographic risk factors and blood samples for serology and genotyping were collected prior to the first biopsy. Serum PSA and hK2 levels were measured using assays previously described (Nam et al. 2000).

### Genotyping

We genotyped two polymorphisms (rs2664155/KL1 and rs198977/KL2) of the KLK2 gene that we previously showed to be positively associated with prostate cancer risk (Nam et al. 2006). Genotyping was conducted using mass-spectrometry-based genotyping analysis using matrix-assisted laser desorption ionization – time of flight (MassArray System, Sequenom Inc., San Diego, California, USA) following the manufacturer’s instructions. Details of the SNPs regarding location, allele type, location and primers have been previously described in detail (Nam et al. 2006).

### Data analysis

Cases were defined as patients with adenocarcinoma of the prostate on repeat biopsy and controls were men with no evidence of cancer (including benign prostatic hypertrophy, high-grade prostate intra-epithelial neoplasia and atypical small acinar proliferation). All baseline risk factors between cases and controls were compared. We ensured that KLK2 genotype frequencies were in Hardy-Weinberg equilibrium when examined in the control population. Serum hK2 levels and KLK2 genotype frequencies were compared between cases and controls. Serum hK2 levels were analysed by quartiles, which were based on the distribution of hK2 levels in the control group. Conventional risk factors together with hK2 levels and KLK2 genotype were examined using multivariable unconditional logistical regression modeling. Both SNPs and serum hK2 levels, the variables of interest in this study, were included in the multivariate model and the inclusion of other variables into the final model was based on significance in univariate analysis or if widely accepted as a risk factor (e.g. family history). We determined the predictive value of our new model by comparing the area under the receiver operating characteristics curve (AUC) of our baseline risk model, which included age, ethnic background, PSA, DRE, past histology, prostate volume, and family history to the AUC after incorporating KLK2 genotype and hK2 level. Bootstrap with 2000 replicates was used to correct the over-fitting bias on the calculation of the AUCs.

We also sought to incorporate hK2 levels and KLK2 genotype into a nomogram to estimate the probability of prostate cancer at the time of repeat biopsy. This nomogram was designed in order to predict all prostate cancers as well as ≥ Gleason 7 prostate cancer. We characterized the clinical effects of this model using decision curve analysis (Vickers & Elkin 2006). This methodology estimates the net benefit for predictive models by summing benefits (true positives) and subtracting the harms (false positives), whereby the latter is weighted using a factor related to the relative harm of a missed cancer in comparison to an unnecessary biopsy. The statistical significance is determined at a critical level of 0.05. Statistical analyses were completed using SAS (Version 9.1, SAS Institute Inc., Cary, NC, USA) the open source software R-2.11.0 (R Development Core Team, 2010) with additional package Design, and S-PLUS (Version 8, Insightful Corporation, Seattle, WA, USA).

## Results

A total of 941 men underwent repeat TRUS guided needle-core biopsy, of which 180 (19.1%) were found to have prostatic adenocarcinoma (cases). Of the patients with cancer, 6 (3.3%), 121 (67.2%), 46 (25.6%) and 7 (3.9%) men had Gleason ≤5, 6, 7 and ≥8 adenocarcinoma, respectively. On univariate analysis, lower prostate volume and non-benign initial biopsy histology (HGPIN and ASAP) were associated with prostate cancer (Table 1). Age, PSA, family history of prostate cancer, ethnicity, urinary symptoms and DRE were not significantly associated with prostate cancer risk at the time of repeat biopsy (Table 1). Serum hK2 levels were significantly higher among cases (mean hK2 = 0.246 ng/mL) than controls (mean hK2 = 0.228 ng/mL, p = 0.02), and the rs198977 KLK2 SNP was significantly associated with prostate cancer (Table 1).

**Table 1:** **Frequency distribution of established and putative risk factors for prostate cancer among cases and controls**

Subgroup		Cases (n = 180), (%)	Controls (n = 761), (%)	***P (χ*** ^***2***^ )*
**Age**	<50	4 (14.3)	24 (85.7)	0.19
	50-60	41 (15.4)	225 (84.6)	
	60-70	87 (20.0)	348 (80.4)	
	>70	48 (22.6)	164 (77.4)	
**PSA (ng/mL)**	<10.0	120 (18.6)	527 (81.4)	0.65
	10.0-20.0	46 (19.7)	188 (80.3)	
	>20.0	14 (23.3)	46 (76.7)	
**TRUS volume (cc)**	<47.0	77 (28.5)	193 (71.5)	<0.0001
	47.0-67.0	51 (21.0)	192 (79.0)	
	67.0-93.1	27 ( 12.7)	185 (87.3)	
	>93.1	25 (11.6)	191 (88.4)	
**Family history**	Negative	152 (18.5)	668 (81.5)	0.23
	Positive	28 (23.1)	93 (76.9)	
**Presence of LUTS**	Negative	104 (21.4)	382 (78.6)	0.067
	Positive	76 (16.7)	379 (83.3)	
**DRE**	Normal	130 (18.6)	569 (81.4)	0.61
	Abnormal	48 (20.1)	191 (79.9)	
**Ethnic background**	Other	3 (9.1)	30 (90.9)	0.074
	Asian	8 (11.9)	59 (88.1)	
	White	147 (19.4)	609 (80.6)	
	Black	22 (25.9)	63 (74.1)	
**Initial Biopsy Pathology**	Normal or BPH	93 (14.8)	537 (85.2)	< 0.0001
	HGPIN	62 (25.6)	180 (74.4)	
	ASAP	25 (36.2)	44 (63.8)	
**rs2664155 (KL1)**	GG	80 (18.4)	354 (81.6)	0.35
	GA	74 (18.5)	326 (81.5)	
	AA	26 (24.3)	81 (75.7)	
**rs198977 (KL2)**	CC	70 (15.8)	374 (84.2)	0.018
	CT	82 (20.9)	311 (79.1)	
	TT	28 (26.9)	76 (73.1)	
**Mean (Median) hK2 Level (ng/mL)**		0. 246 (0.193)	0.228 (0.163)	0.02

*Abbreviations*: TRUS = Transrectal Ultrasound, DRE = Digital Rectal Exam, LUTS = Lower Urinary Tract Symptoms; *Chi-Square Test.

From multivariable analysis of the baseline risk factors, age, TRUS volume, ethnic background and initial biopsy histology were associated with prostate cancer on repeat biopsy (Table 2). Among the two KLK2 SNP variants, KL2 (rs198977) was positively associated prostate cancer at repeat biopsy (OR variant T allele = 1.81, 95% CI: 1.04-3.13, p = 0.049). Additionally, serum hK2 level was associated with prostate cancer (OR = 2.03, 95% CI: 1.4-2.99, p = 0.002, Table 2).

**Table 2:** **Multivariable analysis of KLK2 SNPs and hK2 levels in predicting the presence of prostate cancer at the time of repeat biopsy**

Subgroup		Odds Ratio	95% CI	***P***
**Age**		1.35	1.02 - 1.79	0.049
**PSA (ng/mL)**		1.41	1.02 - 1.96	0.092
**DRE**		1.20	0.80 - 1.80	0.372
**Presence of LUTS**		0.80	0.56 – 1.13	0.205
**TRUS volume (cc)**		0.33	0.24 - 0.45	< 0.0001
**Family history**		1.40	0.86 - 2.28	0.175
**Ethnic background**	Other + Asian	1.00		0.006
	White	2.30	1.15 - 4.62	
	Black	4.01	1.70 - 9.45	
**Initial Biopsy Pathology**	BPH or Normal	1.00		
	HGPIN	1.79	1.22 - 2.62	0.0002
	ASAP	2.62	1.51 - 4.54	
**rs2664155 (KL1)**	GG vs. GA	1.47	0.84 - 2.57	0.218
	GG vs. AA	1.14	0.78 - 1.69	
**rs198977 (KL2)**	TT vs. CT	1.23	0.72 – 2.09	0.049
	TT vs. CC	1.81	1.04 – 3.13	
**hK2 Level (ng/mL)**		2.03	1.4 – 2.99	0.002

The area under the curve (AUC) for the baseline model, which included age, ethnic background, PSA, DRE, past histology, prostate volume, and family history was 0.67. The addition of hK2 levels and the KLK2 SNPs to the baseline predictive model increased the AUC to 0.69 (p = 0.586 for the difference by 2000 bootstrap resamples).

**Figure 1:**
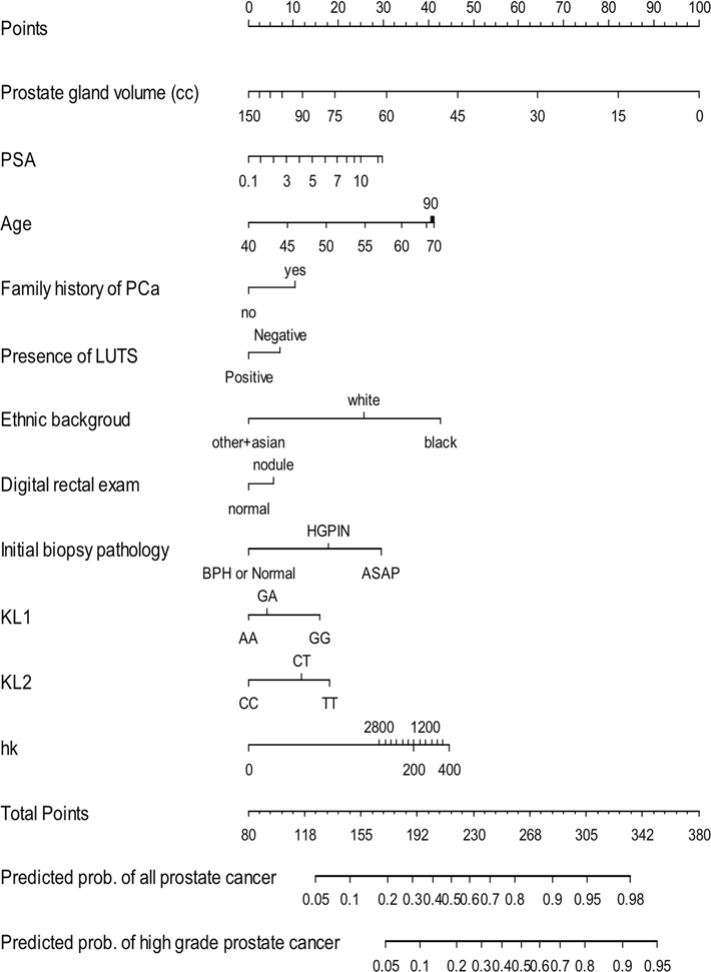
**Nomogram for predicting prostate cancer (PCa) at the time of repeat biopsy.** The nomogram is used by localizing a patient’s position for each variable along its horizontal axis to determine a point value according to the points scale (top axis). The total points corresponds a probability value of having prostate cancer or ≥ Gleason 7 (high grade) prostate cancer. Note: PSA (ng/ml), LUTS = Lower Urinary Tract Symptoms, rs2664155/KL1 (AA = Wild Type; AG = heterozygote; GG = variant), rs198977/KL2 (CC = Wild Type; CT = heterozygote; TT = variant), hk = hK2 level (pg/mL).

**Figure 2:**
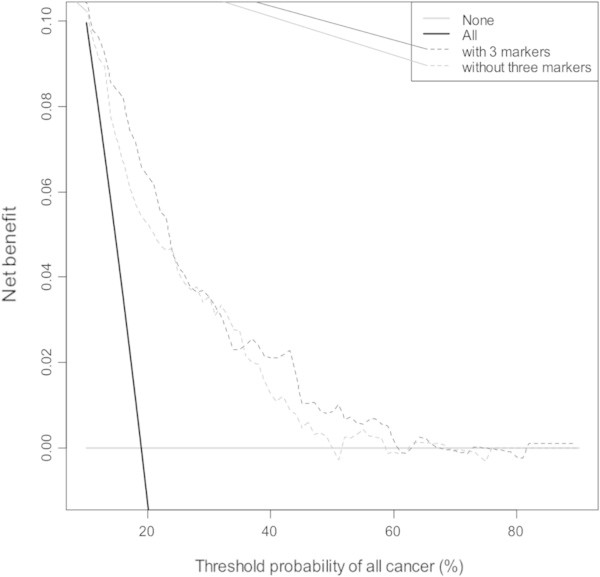
**Decision curve for outcome of any prostate cancer using the three markers studied (rs198977/KL2, rs2664155/KL1 and hK2 level).** The 3 marker strategy (black dashed line) is compared to the baseline model (red dashed line) and to a strategy of biopsying all men (solid grey line) or biopsying no men (solid black line).

The relationships between rs198977 and hK2 levels were examined using autosomal dominant and recessive models (Table 3). The KLK2 rs198977 genotypes were positively associated with hK2 levels (p = 0.02). When combined, the odds ratio for prostate cancer for patients with high hK2 levels and the variant T-allele of rs198977 was 3.77 (95% CI: 1.94-7.32, p < 0.0001), compared to patients with low hK2 levels and the C-allele (Table 4).We incorporated hK2 level and KLK2 SNPs to construct a nomogram for calculating the probability of a prostate cancer, and in particular, for ≥ Gleason 7 prostate cancer on repeat biopsy (Figure 1). Figure 2 shows the decision curve for our full model, including both KLK2 SNPs and hK2 level. The net benefit of the three markers, however, was not superior to alternative strategies, including performing biopsies based on conventional risk factors.

**Table 3:** **Relationship between rs198977 genotype and hK2 levels**

KL2 Genotype	hK2 Level (ng/mL)*	KL2 Genotype	hK2 Level (ng/mL)*
rs198977		rs198977	
**CC (n = 444)**	0.214 ± 0.213	**CC (n = 444)**	0.214 ± 0.213
**CT or TT (n = 497)**		**CT (n = 393)**	0.240 ± 0.241
	0.247 ± 0.243	**TT (n = 104)**	0.271 ± 0.251
***P (between genotypes)***	0.03		0.02

*Abbreviations*: C, Wild-Type Allele; T, Variant Allele. *t-test.

**Table 4:** **Adjusted Odds ratios for prostate cancer detection for each combination of rs198977 Genotype and circulating hK2 levels**

	KL2 Genotype			
	rs198977			
hK2 Level	CC		CT or TT	
	OR (95% CI)	***P***	OR (95% CI)	***P***
**< 0.101**	1.00	-	0.86 ( 0.40 - 1.83)	0.22
**0.101-0.163**	1.51 (0.7 - 3. 2)	0.81	1.49 (0.75 - 2.97)	0.74
**0.163-0.289**	1.42 ( 0.68-2.98)	0.61	3.77 (1.94 - 7.32)	< 0.0001
**>0.289**	1.59 (0.71-3.55)	0.97	2.65 (1.29 - 5.44)	0.026

## Discussion

The purpose of this study was to examine predictors of prostate cancer at repeat biopsy in a prospective fashion with intent to re-biopsy all men with an initial negative biopsy. Among pre-screened men undergoing a repeat biopsy, we have found an association between the KLK2 SNP rs198977 as well as serum hK2 levels and prostate cancer. Serum levels of hK2 were positively associated with rs198977 variants, however, incorporation of these markers to our model did not significantly improve its predictive ability. While the latter highlights a potential lack of clinical utility, hK2 and KLK2 SNPs may still have a discriminatory role, but require further characterization.

Djavan et. al. published a large prospective study of 1050 men with PSA 4 to 10 ng/ml in whom systematic sextant biopsies were performed in all those with negative initial biopsy (Djavan et al. 2000). Of the 820 men with initial negative biopsies, 10% were found to have prostate cancer on repeat biopsy, which was most strongly predicted by percent free PSA and transition zone PSA. The low detection rates of prostate cancer on repeat biopsy was likely due to the exclusion of men with PSA >10. No other novel factors were examined. More contemporary studies have consisted of case series with a focus on PSA and rate of change. While there have been nomograms derived from these studies, they offer conflicting evidence on the role of PSA and its calculated kinetics (e.g. doubling time) in predicting prostate cancer in the repeat biopsy setting (Moussa et al. 2010; Benecchi et al. 2008).

This current study adds to the observation that PSA is a poor marker for guiding which patients require a repeat biopsy. In the setting of repeat prostate biopsy, percent free PSA and PSA density may be useful (Djavan et al. 2000), however, the only established predictors of prostate cancer are the presence of high grade prostatic intraepithelial neoplasia (HGPIN) and atypical small acinar-cell proliferation (ASAP) (Epstein & Potter 2001). Recent studies have shown that ASAP and HGPIN can increase risk of prostate cancer by 42% and 16% respectively in patients undergoing repeat extended core biopsy (Campos-Fernandes et al. 2009). In our analysis of risk factors, we have show that the presence of HGPIN remains a strong predictor of prostate cancer on repeat biopsy, despite the fact its role has been questioned in recent literature (Gallo et al. 2008).

The biology of prostate cancer, particularly, the shared androgen dependence of PSA and hK2, taken together with our previous work adds to the mounting evidence that KLK2 is a prostate cancer susceptibility gene. While it is established that hK2 activates the precursor of PSA, the role of this serine protease in normal and malignant cells is unclear, specifically the mechanism by which the SNPs studied may modulate enzymatic activity and their relationship to serum hK2 levels. In our previous work, the group of patients having the highest quartile of hK2 level and ‘T’ variant of rs198977 (KL2) had the highest risk (OR 13.9, p = 0.0001) (Nam et al. 2003). The variant (TT) genotype of KL2 positively correlated with hK2 levels in this study, whereas the opposite effect had been demonstrated previously (Nam et al. 2006).

Our previous work has established the utility of hK2 levels and KLK2 SNPs in the screening setting (Nam et al. 2000; Nam et al. 2003; Nam et al. 2006). More recently, others have integrated hK2 with intact, total, and free PSA to construct a “kallikrien-panel” that has been shown to improve the prediction of prostate cancer in previously unscreened men with an elevated PSA (>3 ng/mL) derived from the Rotterdam cohort of the ERSPC (European Randomized Study of Screening for Prostate Cancer) (Vickers et al. 2008). Gupta *et al.* applied this panel to men with negative initial biopsies, however, only 40% of men underwent repeat biopsy, due to refusal or a PSA of less than 3.0 ng/mL which may explain the low cancer detection rate of 12% (Gupta et al. 2010). Nevertheless, the addition of the panel containing hK2 to a baseline model appeared to improve the AUC. However, the authors did not control for initial biopsy histology such as ASAP or HGPIN.

The strength of this study lies in a large prospective cohort that was rigorously offered repeat biopsy. We have, for the first time, incorporated hK2 levels with a novel SNP to enhance the prediction of prostate cancer in this setting. Nonetheless, while our nomogram may have clinical utility, the added improvement to this instrument by the addition of hK2 and KL2 SNP was minimal and not statistically significant. In our analysis, the predictive ability of our model only improved minimally from 0.67 to 0.69 and was not significant. Moreover, decision curve analysis did show improved net benefit over the clinical model across most threshold properties, although this is a minimal benefit. Future direction for this work will include incorporating other biomarkers to improve prediction, and validation of this nomogram in other cohorts.

## Conclusions

The KLK2 SNP rs198977 was found to be positively associated with hK2 levels and shown to predict prostate cancer at the time of repeat prostate biopsy. Incorporation of this marker into our model did not significantly improve its predictive ability. Further work is required to characterize the KLK2 gene to potentially improve its clinical utility.
